# Design and Validation of Primer Sets for the Detection and Quantification of Antibiotic Resistance Genes in Environmental Samples by Quantitative PCR

**DOI:** 10.1007/s00248-024-02385-0

**Published:** 2024-05-15

**Authors:** Lizandra Perez-Bou, Alejandro Gonzalez-Martinez, Juan J. Cabrera, Belen Juarez-Jimenez, Belen Rodelas, Jesus Gonzalez-Lopez, David Correa-Galeote

**Affiliations:** 1 Environmental Microbiology Group, Department of Microbiology and Virology, Faculty of Biology, University of Havana, Havana, Cuba; 2 Microbiology and Environmental Technologies Section, Water Research Institute, University of Granada, Granada, Spain; 3 Department of Microbiology, Faculty of Pharmacy, University of Granada, Granada, Spain; 4 Nitrogen Metabolism Group, Zaidín Experimental Station, Spanish National Research Council, EEZ-CSIC, Granada, Spain

**Keywords:** Antibiotic resistance genes, Primer design, Assay optimization, qPCR, Antibiotics, WWTPs

## Abstract

The high prevalence of antibiotic resistant bacteria (ARB) in several environments is a great concern threatening human health. Particularly, wastewater treatment plants (WWTP) become important contributors to the dissemination of ARB to receiving water bodies, due to the inefficient management or treatment of highly antibiotic-concentrated wastewaters. Hence, it is vital to develop molecular tools that allow proper monitoring of the genes encoding resistances to these important therapeutic compounds (antibiotic resistant genes, ARGs). For an accurate quantification of ARGs, there is a need for sensitive and robust qPCR assays supported by a good design of primers and validated protocols. In this study, eleven relevant ARGs were selected as targets, including *aadA* and *aadB* (conferring resistance to aminoglycosides); *ampC*, *bla*_TEM_, *bla*_SHV_, and *mecA* (resistance to beta-lactams); *dfrA*1 (resistance to trimethoprim); *ermB* (resistance to macrolides); *fosA* (resistance to fosfomycin); *qnrS* (resistance to quinolones); and *tetA*(A) (resistance to tetracyclines). The in silico design of the new primer sets was performed based on the alignment of all the sequences of the target ARGs (orthology grade > 70%) deposited in the Kyoto Encyclopedia of Genes and Genomes (KEGG) database, allowing higher coverages of the ARGs’ biodiversity than those of several primers described to date. The adequate design and performance of the new molecular tools were validated in six samples, retrieved from both natural and engineered environments related to wastewater treatment. The hallmarks of the optimized qPCR assays were high amplification efficiency (> 90%), good linearity of the standard curve (*R*^2^ > 0.980), repeatability and reproducibility across experiments, and a wide linear dynamic range. The new primer sets and methodology described here are valuable tools to upgrade the monitorization of the abundance and emergence of the targeted ARGs by qPCR in WWTPs and related environments.

**Supplementary Information:**

The online version contains supplementary material available at 10.1007/s00248-024-02385-0.

## Introduction

The antibiotic resistance phenomenon is one of the biggest issues of concern for human health in the twenty-first century. Controlling the dissemination of antibiotic resistance genes (ARGs) in the environment is a key challenge to guarantee the longevity of the therapeutic capacity of these important pharmaceuticals [1]. Once released into the environment, antibiotic compounds cause the enrichment of antibiotic resistant bacteria (ARB), even in sub-inhibitory concentrations [2]. Therefore, due to the widespread dissemination of ARB, the proliferation of ARGs, and their potential mobilization through horizontal gene transfer, a reliable determination of their occurrence and abundance is required in order to improve the understanding of their dynamics in environmental hotspots. Wastewater treatment plants (WWTPs) are important reservoirs of ARGs via inefficient management or treatment of highly antibiotic-concentrated wastewaters, contributing to the dissemination of both ARGs and ARB to receiving water bodies [3]. A lack of clear guidelines for acceptable levels of antibiotic, ARB, and ARG pollution warrants the design of assays for their quantification in environmental samples to prevent the spread of ARB. In this regard, the necessity to monitor the clinically and anthropogenically relevant ARGs has been highlighted by several governmental entities, i.e., the U.S. Centers for Disease Control and Prevention (https://www.cdc.gov/drugresistance/index.html), the National Aquatic Resource Survey of the U.S. Environmental Protection Agency [4], and the Antimicrobial Resistance Surveillance System of the European Centre for Disease Prevention and Control (https://www.ecdc.europa.eu/en/antimicrobial-resistance).

Currently, ARGs’ monitorization relies on the use of highly accurate molecular-based methods, progressively displacing time-intensive and expensive culture-based approaches [3]. In this respect, quantitative real-time polymerase chain reaction (qPCR) has become the gold standard method for the detection and quantification of ARGs in environmental samples, due to technical advantages such as faster results, more specific detection, and the user-friendly methodology [3]. A plethora of research addressed the abundance of the different ARGs in environmental samples by qPCR, including wastewater [2], activated sludge [5], and wastewater impacted surface water [6], soil samples and manure [7]. In general, the success of a qPCR assay requires appropriately validated primers and correct standards, together with the optimization of the assay performance according to the minimum information for publication of quantitative real-time PCR experiments (MIQE guidelines) [8]. Frequently, these parameters are not optimized enough, which could result in non-specific amplification or under-quantification of the targets leading to inaccurate and imprecise results [9]. In this regard, to obtain the highest accuracy of the abundance measures of a specific target gene during qPCR assays, the proper design of the corresponding primers stands out as the most important factor [10]. Literature regarding quality primer pair design describes several significant properties to consider, mainly, the primer size, the percentage of guanine and cytosine, the lack of formation of secondary structures, and an adequate range of annealing temperatures [8, 11]. However, some developed qPCR assays are inadequately designed and do not meet these key quality criteria [10]. On the other hand, most previous research has been focused on the characterization of individual pathogens or specific groups within a taxon. This approach restricts the design of universal primers enabling the detection and quantification of the broad genetic divergences for the corresponding antibiotic resistance mechanisms [12]. In addition, it is necessary to continuously update the available molecular tools to avoid the miscalculation of ARGs’ abundance in the environment, which could lead to the underestimation of the extent of their dissemination and the potential for their acquisition by previously sensitive bacteria. Environmental monitoring of ARGs using qPCR requires primer sets useful for the analysis of a wide range of target bacteria in different types of environmental samples, and the main parameters that need to be addressed are the assay’s analytical sensitivity and specificity [8]. Therefore, this study describes the development of new primer sets aimed at amplifying a broader diversity of the ARGs *aadA*, *aadB*, *ampC*, *bla*_SHV_, *bla*_TEM_, *dfrA*1, *ermB*, *fosA*, *mecA*, *qnrS*, and *tetA*(A). The selection of these ARGs was based on the reported clinical importance and incremented resistance to the corresponding antibiotics in WWTPs [13, 14]. The enzymes encoded by the *aadA* and *aadB* genes confer resistance to aminoglycosides (gentamicin, hygromycin B, kanamycin, neomycin, spectinomycin, and tobramycin), the sixth most commonly used antimicrobial class in veterinary medicine in Europe [15]. These genes are frequent pathogens with extensive resistance to antibiotics, many of which are detected in municipal wastewaters [16]. The *ampC* gene and the *bla*_SHV_ and *bla*_TEM_ genes encode AmpC beta-lactamases and extended-spectrum beta-lactamases (ESBL), respectively, which can inactivate most broad-spectrum beta-lactam antimicrobials (third-generation cephalosporins, penicillins, and aztreonam), with the exception of cefepime and carbapenems [17]. Similarly, the *mecA* gene encodes for a penicillin-binding protein (PBP2a), which confers resistance to all beta-lactam compounds [18]. The expression of the *dfrA*1 gene inhibits the therapeutic effect of the combination of trimethoprim/sulfamethoxazole. These antibiotics are poorly removed during wastewater treatment [19]. The presence of *ermB* confers resistance to macrolides and it is also frequently associated with resistance to lincosamide and type B streptogramin, resulting in treatment failure to these three antibiotics groups, which inhibit bacterial protein synthesis [20]. The enzyme encoded by the *fosA* gene confers resistance to fosfomycin, an antibiotic amply released into the environment via wastewater, as it is routinely used for the treatment of urinary infections caused by extensively drug-resistant (XDR) Gram-negative bacteria [21]. The *qnrS* gene mediates the resistance to quinolones, an antimicrobial resistance of the highest priority due to its significance in human medicine, particularly in developing countries [22]. This gene is frequently detected in natural environments since most WWTPs only remove a small amount of this antibiotics [23]. Finally, the *tetA* gene class A (*tetA*(A)) is the molecular marker of the resistance of tetracyclines according to its abundance and relationship with anthropogenic inputs [24], whose broad presence in the environment is related to the nearly universal use of tetracyclines in livestock production [25].

The molecular tools and the new qPCR protocols here developed were validated using DNA extracted from different environmental samples, including activated sludge, river sediment, and agricultural soils, ecosystems previously described as hotspots of the dissemination of the targeted ARGs and as environmental reservoirs of ARB [1, 26–28].

## Materials and Methods

### In Silico Design and Validation of Primer Sets

The specific DNA sequences of the target genes *aadA*, *aadB*, *ampC*, *bla*_TEM_, *bla*_SHV_, *mecA*, *dfrA*1, *ermB*, *fosA*, *qnrS*, and *tetA*(A) were retrieved from the Kyoto Encyclopedia of Genes and Genomes (KEGG, https://www.genome.jp/kegg/), including all sequences with an orthology grade > 70% for a given KEGG orthology number (Table 1SI). The KEGG database entries employed for the present study are listed in Table 1. The sequences were aligned using the MAFFT algorithm, and the in silico design of the primers was performed using the Geneious 2021.1.1 software (Biomatters, Auckland, New Zealand). The specificity of the candidate primers over the target regions was assessed by querying the full genome (chromosomes and plasmids) of each of the strains included in this study (listed in Table 2SI), to ensure the absence of non-specific annealing outside of the target DNA fragment. The complete genomes were retrieved from the GenBank DNA database (https://www.ncbi.nlm.nih.gov/genbank/). Finally, the in silico validated oligonucleotides were synthesized by the company Sigma-Aldrich (Merck, Germany).

**Table 1:** KEGG database entries of sequences for each ARGs selected

Gene target	KEGG database entries
*aadA*	K00984: streptomycin 3″-adenylyltransferase
*aadB*	K17881: aminoglycoside 2″-adenylyltransferase
*ampC*	K01467: beta-lactamase class C
*bla*_TEM_	K18698: beta-lactamase class A TEM
*bla*_SHV_	K18699: beta-lactamase class A SHV
*dfrA*1	K18589: dihydrofolate reductase
*ermB*	K00561: 23S rRNA (adenine-N6)-dimethyltransferase
*fosA*	K21253: glutathione S-transferase fosA
*mecA*	K02545: penicillin-binding protein 2 prime
*qnrS*	K18555: fluoroquinolone resistance protein
*tetA*(A)	K08151: DHA1 family, tetracycline resistance protein

**Table 2:** Sequences of the primers designed in this study and their major features

Gene target	Primer ID	Sequence (5′-3′)	Primer length (bp)	Amplicon size (bp)
*aadA*	aadA-336F	CATTCTTGCRGGTATCTTCGAGC	23	215
	aadA-550R	GCACTACATTYCGCTCATCGC	21	
*aadB*	aadB-118F	GACACAACGCAGGTCACATT	20	419
	aadB-536R	GGTGGTACTTCATCGGCATAG	21	
*ampC*	ampC-535F	GTGAAGCCRTCTGGTTTGAG	20	494
	ampC-1028R	GCGACATAGCTACCAAATCCG	21	
*bla*_SHV_	blaSHV-286F	CAGGATCTGGTGGACTAYTC	20	219
	blaSHV-504R	CGCCTCATTSAGTTCCGTTTC	21	
*bla*_TEM_	blaTEM-335F	CGGATGGCATGACAGTAAGAG	21	275
	blaTEM-609R	TTGCCGGGAAGCTAGAGTAAG	21	
*dfrA*1	dfrA-127F	GTMGGSCGCAAGACDTTYGA	20	255
	dfrA-381R	GWARACATCACCYTCTGGCT	20	
*ermB*	ermB-F	GGAACAGGTAAAGGGCAT	18	434
	ermB-R	TCTGTGGTATGGCGGGTAAG	20	
*fosA*	fosA-7F	ACCGGTCTCAATCACCTGAC	20	300
	fosA-306R	GAGGAAGTAGAACGAATCGCC	21	
*mecA*	mecA-1196F	CTTCACCAGGTTCAACTCA	19	370
	mecA-1565R	CCTTGTCCRTAACCTGAATC	20	
*qnrS*	qnrS-F	GCCAATTGYTACGGKATWGAG	21	227
	qnrS-R	GACTCTTTCARTGATGCRCC	20	
*tetA*(A)	tetA(A)-F	TCATGCARCTYGTAGGMCAGG	21	454
	tetA(A)-R	AKCCATGCCMAWCCGTTCCA	20	

### Optimization of PCR Thermocycling Profiles and Construction of Plasmids Standards for ARGs Quantification by qPCR

To optimize the conditions from PCR amplification of the target ARGs, both genomic DNA from selected culture strains carrying the genes and a pool of DNA previously isolated from activated sludge samples were used as templates. The reference bacterial strains used in this study were purchased from the Spanish Type Culture Collection and cultivated in the corresponding media listed in Supplementary Table 2SI. The total DNAs of the five reference bacterial strains were isolated using a FastDNA kit (QBio/MP Biomedicals, LLC, France) according to the manufacturer instructions.

Amplification reactions were implemented in a final volume of 25 μL, comprising 2.5 μL of 10 × DreamTaq buffer (Thermo Scientific, USA), 0.5 μL of True Pure dNTPs (8 mM) (Canvax, Spain), 0.15 μL of each primer (10 μM), 0.125 μL of 5 U/μL DreamTaq Hot Start DNA Polymerase (Thermo Scientific, USA), 0.125 μL of dimethyl sulfoxide (Sigma-Aldrich, USA), 0.0625 μL of 20 mg/mL of bovine serum albumin (New England Biolabs, USA), 19.3875 μL of ultrapure water, and 2 μL of template DNA. The thermocycling profiles of the reactions were optimized for linearity, sensitivity, specificity, repeatability, and reproducibility. The size of the PCR amplification products was checked by electrophoresis in 2% agarose gels.

For the construction of plasmids standards for ARG quantification, the amplicons were purified using the QIAquick PCR purification kit (QIAGEN, Germany). The purified PCR products were ligated into the PCR 4-TOPO vector (Invitrogen, USA) and then used to transform *Escherichia coli* DH5α competent cells according to the specifications of the TOPO TA cloning system (Invitrogen, USA). Subsequently, eight transformant colonies obtained in each reaction were randomly selected to verify the presence of an insert of the right size by PCR, using agarose gel electrophoresis. Finally, four positive plasmids were selected for each ARG to confirm the correct identity of the inserts by means of Sanger sequencing in the facilities of the Genetic Information Unit of the Scientific Instrumentation Center (University of Granada, Spain). Sequences sharing > 98% identity with the corresponding control sequences were subsequently used as standards.

### Environmental Samples and DNA Extraction

This study included the following types of environmental samples: activated sludge, composting sludge, anaerobic digestion sludge, agricultural soil, and river sediment. Two different activated sludge samples were collected from the aeration tank in the secondary treatment step of the biofactories “Churriana de la Vega-Sur” (AS-CHU, UTM coordinates 30N 44509, 13,343) and “Los Vados” (AS-VA, UTM coordinates 30N 39964, 16,334), Granada, Spain. Composting sludge (COM) was collected from sewage sludge composting performed in the set of the environmental complexes Ecoindustria del Reciclado (EIDER) (Guadix, Granada, Spain, UTM coordinates 30N 92646, 30,931). The biomass of anaerobic digestion sludge (AD) employed in this study was collected from an operational bioreactor producing volatile acids from the olive residue (alperujo) from Instituto de la Grasa (Seville, Spain, UTM coordinates 30N 39770, 38,833). The river sediment (SED) was obtained from the Genil River in Granada City, Spain (UTM coordinates 30 N 43940, 13,314), in which the effluents generated in the AS-CHU are discharged. The agricultural soil (SOIL) was retrieved from a farming field irrigated by the Genil River (UTM coordinates 30N 444331, 4,114,407). All samples were immediately frozen after collection and stored at − 20 °C until DNA extraction.

Total DNA from the environmental samples (500 mg) was extracted using the FastDNA-2mL SPIN Kit for Soil and the FastPrep24 apparatus (MP-BIO, USA), according to the manufacturer instructions. Three independent biological replicates were used from each sample. The quality and concentration of the extracted DNA were measured by spectrophotometry using NanoDrop 2000 (Thermo Scientific, USA) and verified by electrophoresis on 1% agarose. DNA samples were stored at − 20 °C for further use.

### Quantification Assays

**Fig. 1:**
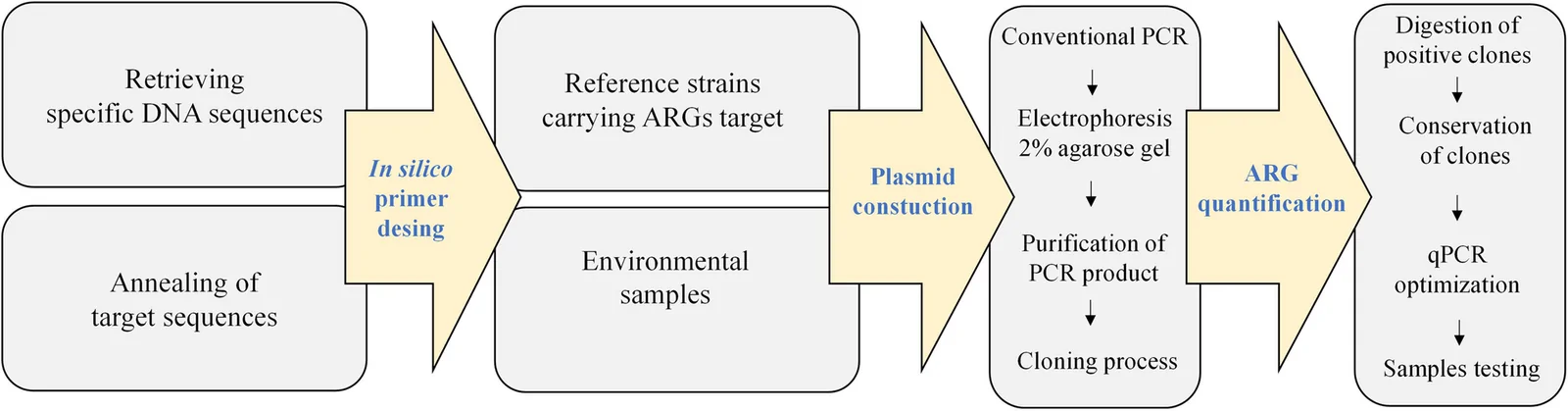
Workflow diagram used in this study for the design and validation of new pairs of primers for ARGs quantification by qPCR

Quantification of the target ARGs in environmental samples was performed by qPCR on a QuantStudio-3 Real-Time -PCR system (Applied Biosystems, USA), using the same reaction conditions as those described in the “Optimization of PCR Thermocycling Profiles and Construction of Plasmids Standards for ARGs Quantification by qPCR” section for conventional PCR, employing SYBR-Green I (0.125 μL, 20 × SYBR Green I (Thermo Scientific, USA)) as dye method for real-time fluorescence monitoring. Serial dilutions of linearized plasmids containing the target genes, ranging from 10^8^ to 10 copies/μL, were used to construct the standard curves for the absolute quantification. The general workflow employed in this study is summarized in Fig. 1.

Samples were run in three independent biological and technical replicates along with non-template controls in two different assays (*n* = 18), to evaluate the repeatability and reproducibility of the qPCR protocols. The number of total copies of the ARGs was expressed per gram of environmental sample, according to the predicted concentration of the corresponding standard amplification plots [29]. In addition, the relative abundance of ARGs was calculated as the normalized ratio of the number of ARGs copies to the corresponding number of genes encoding 16S rRNA copies, which were measured in all samples following a previously described protocol [30].

### Statistical Analyses

The statistical differences among data sets from different samples were analyzed using the non-parametric Kruskal–Wallis and Conover Iman tests (*p* < 0.05 significance level) in XLSTAT v2020 (Addinsoft, USA).

## Results and Discussion

### In Silico Primer Design and ARG Diversity

Sequences of the target ARGs with orthology degrees higher than 70% were retrieved from the corresponding KEGG entries (*aadA*: 96 sequences, *aadB*: 21, *ampC*: 81, *bla*_SHV_: 89, *bla*_TEM_: 81, *dfrA*1: 42, *ermB*: 58, *fosA*: 20, *mecA*: 37, *qnrS*: 15, *tetA*(A): 49) (Table 1SI). Subsequently, alignments were conducted using the MAFFT algorithm implemented in the Geneious Prime software to perform the in silico primer design. This process was made following the recommendations based on coverage and specificity criteria of Dreier et al. [11], the identification of a specific target nucleotide sequence, and the later design of primers that bind exclusively to the target sequences. According to the parameters suggested by Bustin and Huggett [9], the adequate annealing temperatures (50–65 °C), base composition (GC content ranging of 50–60%), length (between 18 and 25 pb), absence of secondary structure, lack of potential hairpin formation, and lack of self-annealing were accomplished. To guarantee the total in silico coverage of the newly designed primers on the template sequences, degenerated bases were introduced into the primers to match all the sequences (maximum 2 degenerations per primer) wherever necessary. The new primer sets guaranteed the total coverage of the template sequences (allowing two ambiguities per primer), generating amplicon sizes between 215 and 494 bp. In addition, the specificity of amplification of the new primers was tested in the 557 chromosomal and plasmid genomes used in this study, without obtaining in silico annealings out of the genetic zones of the genomes previously described as antibiotic resistance coding sequences.

The candidate primers that matched over every targeted sequence and only exclusively over the target regions were considered for further research. The sequences of the new primers designed here and their main features are presented in Table 2. Higher coverages of the newly developed primers for the quantification of the selected sequences of *aadA*, *ampC*, *bla*_SHV_, *dfrA*1, *mecA*, *qnrS*, and *tetA*(A) genes were found, compared to those of all the previously available primers tested in this study (Table 3SI). Additionally, most primers formerly described for the *fosA* gene also presented very low coverages, except those developed recently by Abbott et al. [31], however, the presence of a triplet at the beginning of the forward primer and a higher level of homodimers in the reverse primer are detrimental compared to the new primer set (fosA-7F/fosA-306R) designed in this study. On the other hand, several of the available primers for *aadB*, *bla*_TEM_, and *ermB* presented coverages of 100% over the selected sequences; however, a large number of non-compliances with the recommendations of the MIQE guidelines were found for these primers, i.e., production of secondary structures of the amplicon sequence, high potential to form primer dimers, and triple repeats of the same nucleotide. Therefore, this underlines the necessity to develop new ones that solve those issues, facts that were minimized in the new primers designed. In this sense, Keenum et al. [3] recently reported poor coverages of some of the most common primers used in the literature for the quantification of ARGs, highlighting the necessity to improve the molecular tools currently available for qPCR quantification in order to increase sensitivity and specificity of the assays.

**Table 3:** Thermocycler conditions for quantification of the different ARGs by qPCR

		*aadA*	*aadB*	*ampC*	*bla*_SHV_	*bla*_TEM_	*dfrA*1	*ermB*	*fosA*	*mecA*	*qnrS*	*tetA*(A)
Initial denaturalization		95 °C, 10 min	95 °C, 10 min	95 °C, 10 min	95 °C, 10 min	95 °C, 10 min	95° C, 10 min	95 °C, 10 min	95 °C, 10 min	95 °C, 10 min	95 °C, 10 min	95 °C, 10 min
Amplification (× 35 cycles)	Denaturalization	95 °C, 30 s	95 °C, 30 s	95 °C, 30 s	95 °C, 15 s	95 °C, 15 s	95 °C, 15 s	95 °C, 15 s	95 °C, 30 s	95 °C, 30 s	95 °C, 15 s	95 °C, 30 s
	Annealing	60 °C, 30 s	60 °C, 30 s	60 °C, 30 s	55 °C, 30 s	60 °C, 30 s	55 °C, 30 s	55 °C, 30 s	60 °C, 30 s	50 °C, 30 s	55 °C, 30 s	55 °C, 30 s
	Elongation	72 °C, 40 s	72 °C, 40 s	72 °C, 40 s	72 °C, 40 s	72 °C, 40 s	72 °C, 40 s	72 °C, 40 s	72 °C, 40 s	72 °C, 40 s	72 °C, 40 s	72 °C, 40 s
Melting curve		60 °C–95 °C + 0.15 °C/s. Fluorescence measured each 15 s										

### Optimal PCR Thermocycling Profiles for the Amplification of ARGs Using the New Primer Sets

The specificity of the new primer sets were tested in vitro using as templates genomic DNA of the corresponding reference strains previously described as carrying ARGs in their genomes, as well as environmental DNA pools. After validating different temperatures and times for each of the reaction steps, the thermal profiles that generated better amplification efficiencies are described in Table 3. In all cases, a unique amplification band with the expected amplicon size was observed from both pure cultures (reference strains) and environmental DNA, highlighting the specificity of the molecular tools developed here [10]. An example of the in vitro validation of the specificity of the new primer sets and PCR amplification efficiency is shown in Fig. 1SI.

### Validation of Real-Time Quantitative PCR Assays

The quantification of the gene copy numbers by qPCR is based on the linear relationship between the logarithm of the initial template quantity and the quantification cycle (Cq) value during amplification. A wide linear dynamic range is one of the key performance parameters to achieve in the design of a new qPCR method. This is one of the most important advantages of qPCR assays, since it ensures the accurate quantification of the copy numbers of the target genes, even if the range of abundance spans several logarithmic units [9]. Also, the linearity in the qPCR quantification must be reported through the coefficient of determination (*R*^2^ value) between the Cq values and the logarithm of gene copy numbers [7]. In this respect, the standard curves of the Cq values and the gene abundances here reported showed good linearity in the range from 20 to 2 × 10^8^ gene copies (see details in Supplementary Fig. 2SI)), with *R*^2^ values > 0.990 (Table 4). Hence, the new developed qPCR protocols provided proper fits of the distribution of the data and the accurate estimation of the abundances of the different ARGs within a broad linear dynamic range.

**Table 4:** Equation of the linear regression of standard curves, *R*^2^ value, and amplification efficiency of each qPCR assay

Target gene	Standard curve	*R*^2^	Amplification efficiency
*aadA*	*y* = *− 3.507x* + *34.893*	0.997	100%
*aadB*	*y* = *− 3.4644x* + *31.965*	0.995	94%
*ampC*	*y* = *− 3.5201x* + *33.994*	0.995	92%
*bla*_TEM_	*y* = *− 3.5512x* + *35.579*	0.998	91%
*bla*_SHV_	*y* = *− 3.5431x* + *34.665*	0.998	92%
*dfrA*1	*y* = *− 3.5276x* + *33.713*	0.998	92%
*ermB*	*y* = *− 3.5199x* + *33.689*	0.994	92%
*fosA*	*y* = *− 3.487x* + *33.185*	0.997	94%
*mecA*	*y* = *− 3.5821x* + *34.339*	0.997	90%
*qnrS*	*y* = *− 3.5798x* + *34.293*	0.998	90%
*tetA*(A)	*y* = *− 3.587x* + *35.124*	0.996	90%

Considering that the abundance of ARGs in certain types of samples may be very low, the development of a qPCR assay must be designed to differentiate a low number of copies of a given ARG in a sample from the inherent noise of the method. In this regard, the Cq values of the non-template assays (negative controls) were null or at least 3.3 cycles higher than those of the last dilution of the last standard point; accordingly, no significant background noise was found in these experiments [32]. Besides, it is necessary to calculate the limits of detection (LOD) and quantification (LOQ) of the new qPCR methods as indicators of the quantification accuracy [32]. The LOD values, based on detecting the target sequence at the lowest concentration of the standard curve, ranged 80–120 copy numbers, and, similarly, the LOQ values of the assays corresponded to 4 × 10^4^ gene copies per gram of environmental sample, which reflects the assay’s capacity to precisely quantify the target genes expressed as gene copies abundance per gram of matrix.

The efficiency values of the different qPCR assays described here ranged between 90 and 100% (Table 4); therefore, good performances of the developed methods were found, according to Keenum et al. [3]. In this respect, variable amplification efficiencies have been reported in the quantification range for several ARGs, including *bla*_CTX-M_ (95.3%), *bla*_TEM_ (107.4%), *bla*_OXA1_ (92.1%), *ermB* (91.3%), *tetA* (95.4%), *sul*1 (95.8%), *sul*2 (83%), *dfrA*1(88.5%), and *dfrA*12 (99.4%) [7]. The repeatability and reproducibility were determined by comparing the Cq values among analytical replicates in a given qPCR assay and those obtained in the different qPCR assays. In this regard, the intra-analytical deviations of the standards among all qPCR assays were very low (average Cq value = 0.345, ranging from 1.164 to 0.024 cycles). Similarly, the mean analytical deviations among different qPCR experiments were 1.01 cycles (ranging from 0.558 to 1.316).

Finally, the specificity of the qPCR methods was also determined by analyzing the amplicon products after performing the assay [33]. In all cases, the melt curves displayed a single sharp peak and were shoulderless (except for the *ermB* gene which specificity was confirmed by Sanger sequencing), indicating that the amplicons obtained were free of unspecific products, highlighting the reliable amplification of the target ARGs (Fig. 3SI). Also, the verification of the melting temperature of the amplicons from environmental samples was compared with the expected peak of melting temperature obtained for the amplification products of the standards. Both melting temperature peaks were equivalent, confirming the specificity of primer annealing previously observed in the assays for the optimization of the PCR conditions. Therefore, these qPCR methods showed a proper level of analytical repeatability and reproducibility, confirming their effectiveness to quantify several ARGs reliably and accurately.

### Total Quantification of ARGs in Environmental Samples

**Fig. 2:**
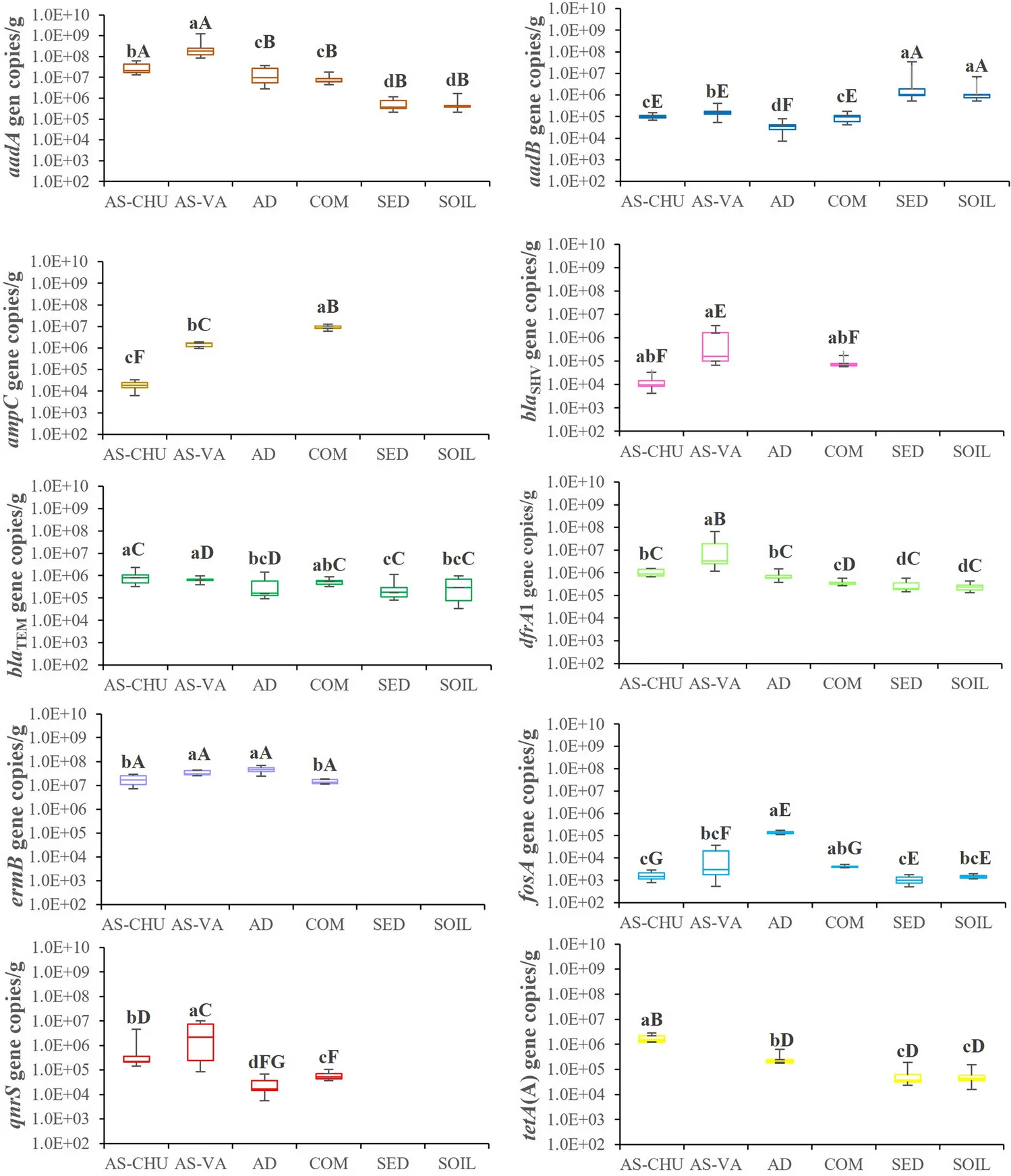
Total abundance of ARGs in the six environmental samples determined as gene copies/g of environmental sample in two independent qPCR reactions (*n* = 18). According to the Kruskal–Wallis and Conover-Iman tests (*p* < 0.05), different lowercase letters indicate significant differences among environmental samples for a given ARG, and different capital letters indicate significant differences among ARGs for a given environmental sample

Developing new qPCR methods requires detecting and quantifying the actual occurrence of a given population in complex DNA from different environmental samples, including rare, cryptic, and elusive genes [34]. For that purpose, the efficiency of the primers, the qPCR conditions, and the effects of different matrices were validated in six natural and engineered environmental samples to show the right level of compliance with the critical considerations that need to be addressed for the validation of the design of new primers and the establishment of newly developed qPCR approaches. The total abundance of the ARGs detected in this study is presented in Fig. 2. The genes *aadA*, *aadB*, *bla*_TEM_, *dfrA*1, and *fosA* were prevalent in all samples. On the other hand, the *ampC*, *bla*_SHV_, *ermB*, *qnrS*, and *tetA*(A) genes were only detected in some samples, suggesting that these ARGs are rare in the different environments analyzed. Finally, the *mecA* gene was not measurable in any samples. According to these results, high ARGs’ detection frequencies (ranging from 70 to 100%) were found in the environmental samples using the de novo primer sets and qPCR methodologies proposed in this research, except for the *mecA* gene. The widespread occurrences and ubiquitous distribution of ARGs for the most used antibiotics (beta-lactams, fluoroquinolones, tetracyclines, macrolides, and sulfonamides) in natural and engineered ecosystems have been identified as a severe public health concern [27], a fact widely attributed to the subminimum concentrations of antibiotics that reach these ecosystems [35].

The total abundances of ARGs among all the samples oscillated from 10^3^ to 10^8^ copies/g of environmental sample. Generally considered, the most abundant ARG was the *aadA* gene (average value 2.81 × 10^7^ copies/g), followed by *ermB* (2.75 × 10^6^ copies/g), *dfrA*1 (2.75 × 10^6^ copies/g), *ampC* (1.90 × 10^6^ copies/g), *aadB* (1.18 × 10^6^ copies/g), *qnrS* (8.10 × 10^5^ copies/g), *bla*_TEM_ (5.45 × 10^5^ copies/g), *tetA*(A) (3.65 × 10^5^ copies/g), *bla*_SHV_ (1.33 × 10^5^ copies/g), and, finally, *fosA* (1.35 × 10^4^ copies/g). According to the Kruskal–Wallis and Conover-Iman tests (Fig. 2), *ermB* and *aadA* were the most abundant genes in all the samples in which these ARGs were detected. The abundances of *aadB*, *bla*_TEM_, *dfrA*1, and *tetA*(A) genes presented a middle prevalence, and the lowest abundances were statistically found for *bla*_SHV_, *fosA*, and *qnrS*. Finally, the numbers of gene copies of *ampC* were highly variable among samples.

### Relative Abundance of ARGs in Environmental Samples

**Fig. 3:**
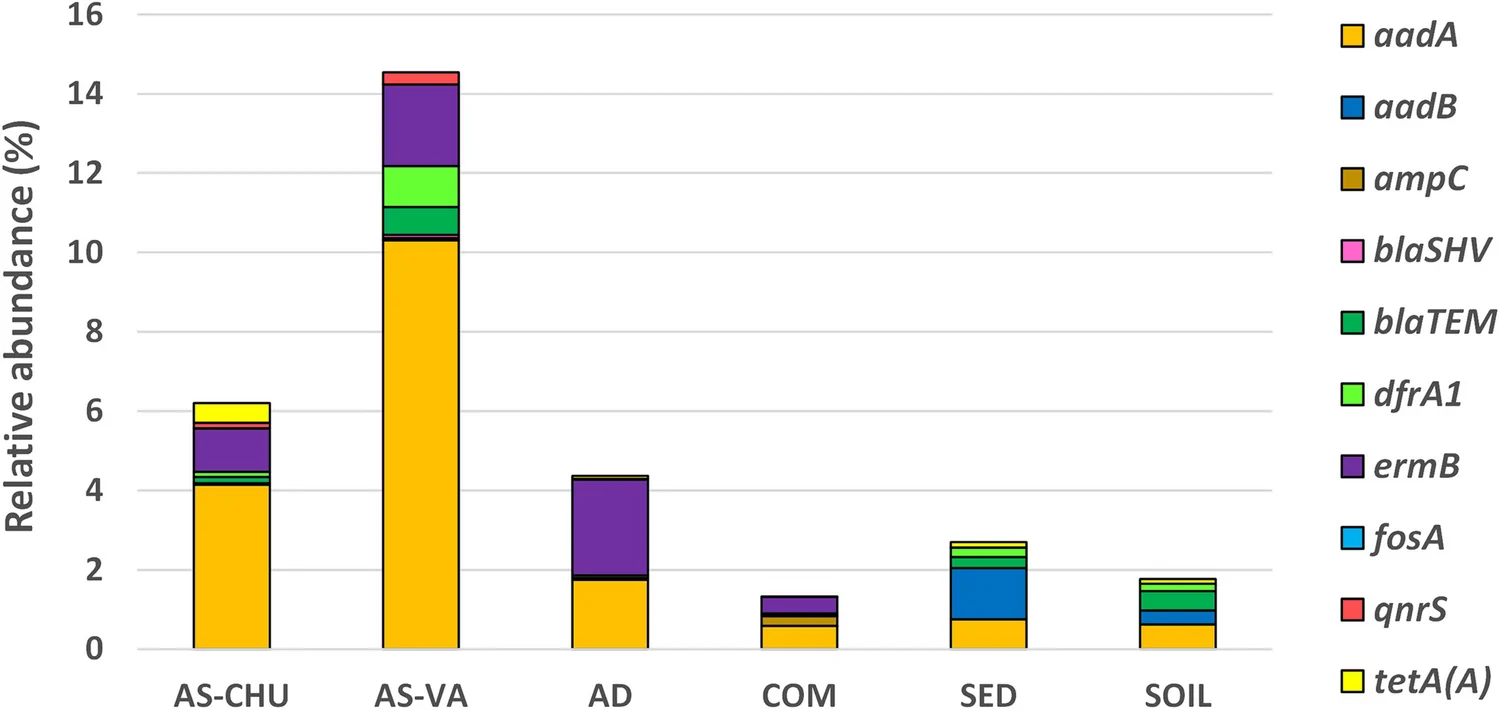
Relative abundance of different ARGs (copies ARG/copies bacterial 16S rRNA) in the six environmental samples determined by qPCR (*n* = 18)

Figure 3 and Table 4SI display the normalized ratio of ARGs copies to 16S rRNA copies (see details of 16S rRNA values in Fig. 4SI).

The highest relative abundance of ARGs for aminoglycosides (*aadA*, average 3.02%) and macrolides (*ermB*, 1.00%) observed in this study is in agreement with those previously found in different WWTPs, anaerobic digestates, livestock manure, and riverine ecosystems [26, 36, 37]. The high prevalence of the *aadA* gene in the six samples regardless of their origin could be linked to the fact that streptomycin has been amply used in animal husbandry and plant disease control since the late 1950s [38], which could have had a strong impact in the development and dissemination of aminoglycoside resistance in the natural environment. However, the use of streptomycin as a first-line antibiotic for tuberculosis treatment makes mandatory to avoid the dissemination of the *aadA* gene within pathogens and other sensitive bacteria [39]. Similarly, the *ermB* gene is one of the most abundant antibiotic-resistant genes within the bacterial communities of wastewater [38], whose dissemination is linked to bacteriophages via transduction, a prevalence that has been connected to the common clinical use of macrolides [37].

The low relative abundances of *aadB*, *ampC*, *bla*_SHV_, *bla*_TEM_, *dfrA*1, *qnrS*, and *tetA*(A) genes suggests that these ARGs are not the dominant resistance mechanisms in the bacterial communities here analyzed. Similarly, low relative abundances of these genes have been described in activated sludge from different WWTPs, anaerobic digestate, compost, riverine sediments, and agricultural soil samples for the *aadB* [37, 40], *ampC* [14, 41], *bla*_SHV_ [14, 42], *bla*_TEM_ [6, 14, 43], *dfrA*1 [3, 43, 44], *qnrS* [2, 45], and *tetA*(A) genes [41, 44]. However, although these ARGs were detected in low relative abundances, their common occurrence implies a loss of the therapeutic effects of some of the most employed antibiotics, resulting in treatment failures in both developed and developing countries, and could be important agents in the exacerbation of antibiotic resistance emergence. Ultimately, although all these genes were detected in low relative abundances in the samples analyzed here, monitoring their occurrence in the environment is essential to prevent their overdispersion, which will increase antibiotic resistance emergence irretrievably.

Finally, low relative abundances of *fosA* and the null presence of *mecA* genes were found in the six environmental samples here analyzed. Although only a few studies have determined the occurrence of the *fosA* gene in environmental samples, its scarce detection is in agreement with the previous results found in isolated bacteria [46] and in different environmental samples [47]. The prevalence of *fosA* in the environment supposes the proliferation of its main reservoirs, which are the well-recognized human pathogens *Klebsiella* spp., *Enterobacter* spp., and *Serratia marcescens* [48]. Besides, the absence of *mecA* gene in the different environmental samples is in agreement with the previous report of Shoaib et al. [49], However, a starting high prevalence of methicillin-resistant bacteria in environmental samples, mainly WWTPs, has been addressed in recent years [50]. Therefore, monitoring of both ARGs needs to be conducted and included in the framework for surveillance in natural environments to fill the gaps in the knowledge of the prevalence in environmental samples of *fosA* and *mecA* encoding resistance to therapeutic compounds essential to fight bacterial infections.

## Conclusions

In this study, eleven new primer sets targeting *aadA*, *aadB*, *ampC*, *bla*_SHV_, *bla*_TEM_, *dfrA*1, *ermB*, *fosA*, *mecA*, *qnrS*, and *tetA*(A) genes were designed in silico fulfilling the strict requisites recommended for the proper development of primers aimed for qPCR, providing improved designs and higher coverages compared to most currently available primers. The validation of the new primer sets and qPCR protocols showed target specificity and high PCR efficiency, ample linear dynamic range, analytical sensitivity with low LOD and LOQ values, repeatability, and reproducibility of the assays, demonstrating their robustness even in samples carrying very low target DNA concentrations. The reliability of the new primers and qPCR protocols was also successfully validated in environmental samples, including river sediment, agricultural soil, activated sludge, compost, and anaerobic digestate. The abundance trends of ARGs differed among samples, highlighting the prevalence of *aadA* and *ermB* genes in all of them. Low relative abundances of *aadA*, *bla*_SHV_, *bla*_TEM_, *dfrA*1, *fosA*, *qnrS*, *tetA*(A) genes and a null-presence of *mecA* gene were found. Particularly, the importance of these ARGs in antibiotic resistance emergence combined with the high occurrence of *aadA* and *ermB* genes confirmed the importance of WWTPs as hotspots of ARB dissemination. The development of effective and reliably new qPCR methods represents an outstanding contribution to quantifying the abundance of the ARGs and can provide information about the occurrence of ARB useful to propose new politics that minimize the emergence of antibiotic resistances. Hence, the improved primer sets developed in this study could be considered valuable tools to accurately monitor the ARGs in the environment as the first step to ameliorate antibiotic resistance phenomena.

## Supplementary Information

Below is the link to the electronic supplementary material.

Supplementary file1 (XLSX 5963 KB)
